# All change, 2025

**DOI:** 10.1002/clt2.70063

**Published:** 2025-05-22

**Authors:** Clive Grattan, Jean Bousquet

**Affiliations:** ^1^ St John's Institute of Dermatology Guy's Hospital London UK; ^2^ Odense University Hospital Odense Denmark; ^3^ Institute of Allergology Charité – Universitätsmedizin Berlin Berlin Germany; ^4^ Fraunhofer Institute for Translational Medicine and Pharmacology ITMP, Allergology and Immunology Berlin Germany

**Keywords:** Clinical and Translational Allergy

Professor Oliver Pfaar took over as Editor‐in‐Chief of Clinical and Translational Allergy (CTA) in April 2025 with Professor Maria Escribese as deputy editor. The journal was founded by Professor Jan Lötvall in 2011 when open access publication of scientific papers on the web to a worldwide readership without restriction to subscription journals was an innovation. Dr Clive Grattan was appointed in 2012 to lead the development of this new initiative of the European Academy of Allergy and Clinical Immunology (EAACI). Professor Jean Bousquet was appointed Co‐editor in Chief in 2016.

The primary aim of CTA is to communicate applied science and clinical research in the field of allergy to daily clinical practice in the English language. The most frequently published article types are original articles, reviews, position articles and letters to the editor. The impact factor has increased from 3.239 at launch in 2016 to 4.6 in 2023. Publication impact is also reflected in altmetrics scores and social media dissemination. The number of manuscript submissions has increased steadily, with a year‐on‐year increase in 2023 of 8.8%. The acceptance rate of 34.9% in 2023 was comparable to other Allergy and Clinical Immunology journals. Submitted and published manuscripts come from across the globe. CTA migrated from BioMed Central to Wiley in 2021 to align with its two sister journals in the EAACI portfolio; Allergy and Paediatric Allergy and Immunology. Having a single publisher has facilitated the transfer of submitted manuscripts between the EAACI journals via Editor Driven Referral. This represented 29% of total submissions to CTA in 2023. The number of accesses and downloads has increased year‐on‐year.

The cost of open access publishing falls to the author or institution rather than the journal owner and its publisher but, in return, the copyright belongs to the copyright holder who is free to read, share and download their work immediately on publication. Different schemes are available to support the cost, including contracts between publishers, funders and research institutions. Waivers or discounts on the article processing charges (APC) are available through Wiley for low‐income countries. EAACI offers discounted APCs for members.

Highly cited early publications in CTA including Diagnostic tools in Rhinology EAACI position paper (2011), mechanisms of allergen‐specific immunotherapy (2012), diagnosis and management of non‐IgE mediated cow's milk allergy in infancy – a UK primary care practical guide (2013), fungal allergy in asthma – state of the art and research needs (2014) and the role of IL‐33 and mast cells in allergy and inflammation (2015) showpiece the breadth and quality of its content. Emerging technologies for predictive medicine in rhinitis and asthma across the life cycle have been an important innovation in generating large amounts of real world data in rhinitis, rhinosinusitis and asthma by engaging patients and their clinicians through mHealth apps.^1^, ^2^ Adapting to the natural environment and promoting a healthy lifestyle should be prioritized by implementing programmes to reduce the burden of respiratory disorders and other non‐communicable diseases resulting from urbanisation and loss of biodiversity leading to long term immune dysfunction.^3^ COVID‐19 had a profound effect on worldwide populations and health policies, including mass vaccination. Could differences in diet of different geographic populations have been partly responsible for differences in death rates between and within countries based on angiotensin converting enzyme 2 activity and antioxidant properties of some foods?^4^ Controversies about COVID vaccination and risk of allergy to excipients, including polyethylene glycol and polysorbates, contributed to vaccine hesitancy but these early concerns were mainly not realized.^5^ Guidance on the diagnostic workup for suspected allergy and decision points for vaccination during the COVID‐19 pandemic played an important role in providing clarity to clinicians and patients with a history of prior polyethylene glycol and/or polysorbate allergy.^5^ From vaccination to immunotherapy, finding the D816V *KIT* variant in peripheral blood of patients undergoing monospecific venom immunotherapy (VIT) was a significant predictor of systemic adverse events during honeybee VIT and a significant predictor of VIT failure after completing wasp VIT.^6^ The introduction of targeted monoclonal antibodies and JAK inhibitors has given relief to many patients with severe atopic dermatitis but modifying underlying immune events may have other potential benefits, such as a decrease in food specific IgE in atopic dermatitis children treated with dupilumab although it was not possible to conclude that reduction of food specific IgE led to tolerance in this preliminary report.^7^ Repurposing a well‐established drug to manage allergic disease outside its original indication is illustrated by a potential role for omalizumab in food allergy management and its recent approval for this new indication by the Federal Drug Administration.^8^ Comparison of interventions remains a key tool to advance best contemporary allergy practice, as illustrated by a recent retrospective comparison of the benefits to infants with cow's milk protein allergy managed with the milk ladder or early oral immunotherapy.^9^ Publication of well designed, adequately powered, original research will continue to enhance good clinical care of patients.

The outgoing editors wish Professor Pfaar and the new editorial team every success in creating new opportunities and directions for the journal in 2025 and beyond. We especially want to thank our hardworking and highly dedicated editorial board, reviewers and authors without whom CTA could not have developed and thrived over the last 14 years.

## Author contributions

**Clive Grattan**: Writing—original draft, conceptualization, writing—review and editing. **Jean Bousquet**: writing—review and editing.

## CONFLICT OF INTEREST STATEMENT

The authors are both immediate past Co‐Editors‐in‐Chief of Clinical and Translational Allergy.
